# Ferroelectric-like metallic state in electron doped BaTiO_3_

**DOI:** 10.1038/srep13207

**Published:** 2015-08-20

**Authors:** J. Fujioka, A. Doi, D. Okuyama, D. Morikawa, T. Arima, K. N. Okada, Y. Kaneko, T. Fukuda, H. Uchiyama, D. Ishikawa, A. Q. R. Baron, K. Kato, M. Takata, Y. Tokura

**Affiliations:** 1 Department of Applied Physics and Quantum-Phase Electronics Center (QPEC), University of Tokyo, Hongo, Tokyo 113-8656, Japan; 2 RIKEN Center for Emergent Matter Science (CEMS), Wako 351-0198, Japan; 3 Department of Advanced Materials Science, University of Tokyo, Kashiwa 227-8561 Japan; 4 Syncrotron Radiation Research Unit, JAEA/SPring-8, Sayo, Hyogo 679-5148, Japan; 5 Materials Dynamics Laboratory, RIKEN SPring-8 Center, Sayo, Hyogo 679-5148, Japan; 6 Research and Utilization Division, JASRI/SPring-8, Sayo, Hyogo 679-5198, Japan; 7 Structural Materials Science Laboratory, RIKEN SPring-8 Center, Sayo, Hyogo 679-5148, Japan

## Abstract

We report that a ferroelectric-like metallic state with reduced anisotropy of polarization is created by the doping of conduction electrons into BaTiO_3_, on the bases of x-ray/electron diffraction and infrared spectroscopic experiments. The crystal structure is heterogeneous in nanometer-scale, as enabled by the reduced polarization anisotropy. The enhanced infrared intensity of soft phonon along with the resistivity reduction suggests the presence of unusual electron-phonon coupling, which may be responsible for the emergent ferroelectric structure compatible with metallic state.

Electric polarization and its coupling with other electronic degrees of freedom in ferroelectrics offer a fertile ground for realizing unconventional quantum phases with unique functionalities. Recent researches uncovered that versatile magnetically/structurally driven ferroelectric phases with magnetoelectric and electromechanical phenomena can be invoked by harnessing the interplay between polarization and spin or crystal-lattice degrees of freedom^1^,^2^,^3^. For examples, the spiral magnetic ordering in Mott insulators often exhibits ferroelectric phases with giant static/dynamical magnetoelectric effects^4^,^5^,^6^,^7^,^8^. So far, the design principle to find a new ferroelectric phase has been implicitly limited to insulators, but the concept might be generalized to metals, in which the ‘polarization’, or equivalently, cooperative polar lattice distortion is coupled to mobile charge. In this context, electron doped ferroelectrics may be unique candidates to study the interplay between conduction electron and polarization.

The perovskite BaTiO_3_ is a prototypical ferroelectric, which is currently utilized in a variety of electronic devices including capacitors, thermistors and nonlinear optical crystals. It shows versatile thermally-induced ferroelectric phases with the macroscopic electric polarization parallel to 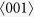, 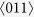 and 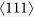 axes as characterized by tetragonal, orthorhombic and rhombohetral crystallographic symmetries, respectively (Fig. 1). (Note that the crystal axes are denoted here in cubic notation.) Although pristine BaTiO_3_ is a band insulator with a gap of about 3.2 eV, it can be turned into a semiconductor or metal by doping conduction electrons into Ti-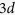 band by means of chemical substitutions or introduction of oxygen vacancies^9^,^10^,^11^,^12^. Specifically, the dilutely doped systems with electron concentration (*n*) less than 1 × 10^20^ cm^−3^ are semiconducting, and exhibit successive ferroelectric-like phase transitions, which manifest as discontinuous changes in resistivity^12^. On the other hand, the heavily doped systems are metallic down to low temperatures and the resistivity curve shows more moderate temperature dependence without discontinuous jumps. Figure 2(a) exhibits the typical profile of resistivity for a heavily doped single crystal with *n* = 1.9 × 10^20^ cm^−3^. Here, we partially substituted Ba-ions with La ions for electron doping (See Methods). The resistivity shows a metallic behavior, except a cusp-like anomaly around 260 K and a small upturn due to weak localization below 70 K.

Figure 2(b) displays the temperature dependence of Hall coefficient (*R*_H_). *R*_H_ exhibits minimal temperature dependence above 260 K, but steeply decreases in absolute value below 260 K. Such a change in the *R*_H_ suggests the reconstruction of Fermi surface at 260 K, which cannot be understood in the scheme of rigid band structure in doped n-type semiconductor. Similar behavior was also observed in the BaTiO_3–*δ*_ with *n* = 1.6 × 10^20^ cm^−3^ 10,12. The non-monotonic temperature dependence of charge transport implies the presence of structural phase transition into an unusual ferroelectric-like state, but the crystal structure in this enigmatic metallic system remains unexplored. In this study, to clarify the underlying interplay between conduction electrons and polarization, we have investigated the crystal structure and electron-lattice coupled dynamics in electron doped BaTiO_3_ by means of synchrotron x-ray scattering, electron diffraction, and infrared optical spectroscopy.

We analyzed the crystal structure by means of single-crystalline synchrotron x-ray scattering and convergent-beam electron diffraction (CBED) on the electron-doped BaTiO_3_, (Ba_0.97_Sr_0.03_)_0.98_La_0.02_TiO_3_. The structural phase transition manifests itself as the splitting of fundamental Bragg reflections. Typical profiles of (400) reflections in the powder diffraction pattern are shown in Fig. 2(c). The peak splits below 350 K, indicating that the crystallographic symmetry is no longer cubic. We show the *d*-spacings of the (400)-reflections as a function of temperature in Fig. 2(d). The *d*-spacing of these two peaks suddenly bifurcates below 350 K. Moreover, the *d*-spacing of higher lying peak again exhibits a discontinuous change at 260 K, which nearly coincides with the cusp-like anomaly in temperature dependence of resistivity and Hall coefficient. These results indicate the presence of successive structural phase transitions at 350 K (=*T*_S1_) and 260 K (=*T*_S2_).

We further explored Bragg reflections in a wide reciprocal space region using the single-crystal x-ray oscillation photographs. Similar peak splitting is identified in other fundamental reflections, but no superlattice reflections are detected within the experimental accuracy. On the basis of collected reflections, the crystallographic symmetry between *T*_S1_ and *T*_S2_ is found to be tetragonal with the space group of either centrosymmetric P4/mmm or noncentrosymmetric P4mm. To distinguish these two candidates, we performed the CBED measurement, which can detect broken symmetries more sensitively than the ordinary x-ray diffraction owing to the dynamical diffraction effect^13^.

Figure 3(a) shows the zeroth-order Laue zone reflections of the CBED pattern taken at 290 K. The CBED pattern with the incident beam parallel to [001] axis exhibits the 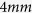 symmetry, that is, a 4-fold rotation symmetry around [001] axis and two mirror symmetries respectively perpendicular to [100] and [110] directions. Conversely, as exemplified in Fig. 3(b), the CBED pattern with the incident beam parallel to [100] axis does not exhibit a mirror symmetry perpendicular to [001] axis, while a mirror symmetry perpendicular to [010] axis is maintained. This is not consistent with the centrosymmetric P4/mmm symmetry, but rather agrees with the noncentrosymmetric P4mm structure, which is compatible with the presence of polar axis (electric polarization) along [001] direction. Given the polar crystallographic symmetry, we refined the single crystalline synchrotron x-ray diffractogram and analyzed the crystal structure with reliability factors *R* = 2.10% and *R*_*wp*_ = 2.49% (Supplemental Information 1). The detail of structural parameters are listed in Table 1.

At temperatures below *T*_S2_, characteristic diffuse scattering emerges near fundamental reflections, in addition to the aforementioned peak splitting. Figure 3(d) exemplifies an x-ray oscillation image around the fundamental Bragg reflection at (009). One can see butterfly-shaped diffuse scattering extending along 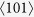 direction, in contrast with the Bragg reflection in tetragonal phase as shown in Fig. 3(c). The spatial correlation length estimated from both the width of diffuse scattering and the dark-field transmission electron microscope image is of the order of 10 nm (Supplementary Information 2). This suggests that the polar structure is heterogeneous on nanometer scale and the macroscopic polar axis points to a certain direction in-between 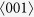 and 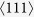 axis within 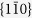 plane as schematically shown in Fig. 1. We note that such a nanometer-scale heterogeneous polar structure with characteristic butterfly-shaped diffuse scattering has not been identified in pure BaTiO_3_, but is rather a common feature of the polar embryo structure in “frustrated”/relaxor ferroelectrics with giant permittivity and piezoelectric response^14^,^15^,^16^. Specifically, at the morphotropic phase boundary in canonical piezoelectrics Pr(Zr_1–*x*_Ti_*x*_)O_3_, the tetragonal phase thermally turns into the frustrated ferroelectric phase with polar embryo structure at lower temperatures, similar to the series of structural transformations in the present system. This low-temperature polar structure appears to be of monoclinic symmetry in a macroscopic scale, but is complex in a microscopic scale. So far, a variety of scenarios including the nanometer scale coexistence of tetragonal-, rhombohedral- and monoclinic-domains have been proposed as plausible microscopic models, but their relevance is still under intensive debate^16^,^17^,^18^,^19^,^20^,^21^,^22^. Regardless of specific models, it is commonly recognized that the “directional frustration” of electric polarization (*P*) driven by the critical competition between the rhombohedral phase (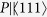) and tetragonal phase (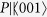) significantly reduces the uniaxial anisotropy, leading to the nanoscale complex domain structures. Since pure BaTiO_3_ and its lightly doped analog with highly insulating state rarely exhibit such a frustrated ferroelectric phase, aside from the metastable state under the intense electric field^23^, it is clear that the introduced conduction electrons play a crucial role in the unique structural phase transitions of the present system. Henceforth, we term this frustrated ferroelectric phase below *T*_S2_ the ‘monoclinic’ phase.

To clarify the entanglement between conduction electrons and electric polarization, we have investigated the charge-lattice coupled dynamics using the infrared spectroscopy. Figure 4(a) is a magnified view of the optical conductivity spectra below 0.04 eV in the paraelectric phase. Two broad peaks and one sharp peak are identified around 7, 15 and 23 meV, respectively. Referring to the infrared phonon modes for BaTiO_3_^24^,^25^, the sharp peak at 23 meV is assigned to the phonon governed by the Ba motion (Last mode), while the broad peaks around 7 and 15 meV can be the polarization relaxation mode (central mode) or Slater mode (soft phonon). Figure 4(b) shows the temperature dependence of optical conductivity spectra. The spectral intensity is significantly enhanced as temperature decreases, while the typical energy scale of the peaks barely changes. Below 100 K, the two peaks combine into, nearly, a single peak. Henceforth, we do not distinguish these two modes and regard them as a soft phonon. In order to quantify the temperature dependence of its spectral intensity, we fitted the spectra with the model of a damped harmonic oscillator coupled to a Debye mode (Supplement Information 3). Figure 4(c) shows the temperature evolution of spectral intensity for the soft phonon. The spectral intensity monotonically increases with decreasing temperature in the whole temperature range. Specifically, it is steeply enhanced below *T*_S2_ and nearly doubles at the lowest temperature. Moreover, the width of soft mode remains as large as 20 meV even at 10 K. The overdamped character and temperature-insensitive energy of the soft phonon well below the transition temperature as well as its enhanced spectral intensity are rarely observed in conventional ferroelectrics. Indeed, in pristine BaTiO_3_, the soft phonon usually exhibits a hardening below the ferroelectric transition temperature while reducing its overdamped character in accord with the growth of ferroelectric order^26^,^27^. This suggests that the delocalized conduction electrons play an important role in the polarization dynamics.

This scenario is further supported by the nonresonant inelastic x-ray scattering (IXS) spectra. Figure 5(a) displays the IXS spectra at various reduced wave vectors (*q*) along (110) in the paraelectric phase taken at 350 K. Here, phonons with polarization parallel to [001] direction, that is, the transverse phonon is discernible due to the nearly orthogonal relation between the polarization vector and *q*. The IXS spectrum at *q* = 0.08 shows two intense peaks at 5 and 23 meV due to the transverse acoustic (TA) phonon and Last mode, respectively. Moreover, the soft mode manifests itself as a broad continuum with significantly reduced scattering intensity around 10 meV. The mode around 28 meV with broad peak width may be attributed to the multiphonon band or infrared inactive optical phonon. We note that a similar intensity profiles of phonons are observed in the perovskite-type ferroelectrics Sr_1–*x*_Ba_*x*_MnO_3_^28^, supporting our mode assignment. To derive the energy of each mode, we assumed the damped harmonic oscillators to reproduce spectra. The energy dispersion of each mode is summarized in Fig. 5(b). The energy of soft mode appears to decrease with approaching Γ-point as often seen in various perovskite-type ferroelectrics. Figure 5(c) displays the temperature dependence of IXS spectra at (0.08 0.08 5). The IXS intensity exhibits minimal temperature dependence around 10 meV, in contrast with the large temperature dependence of infrared spectra. In general, the scattering intensity of nonresonant IXS is governed by that of phonons, while the intensity of infrared spectra is sensitive to both electronic excitations and infrared active optical phonons^29^,^30^,^31^. Therefore, the enhanced infrared intensity of soft phonon suggests the presence of electronic excitation, which is strongly coupled to the soft phonon.

One may think of the possibility of a multiphonon side band of conduction electrons (Drude) response, stemming from the underlying longitudinal phonon mode. However, this is not likely, since the longitudinal mode with the lowest energy is located around 22 meV, as inferred from the spectra of loss function shown in Fig. 4(d). A more plausible scenario is the direct interaction between the doped electrons and soft phonon. It is well known that the interaction between the infrared active optical phonon (transverse optical mode) and underlying electronic excitation manifests itself as an enhancement of infrared spectral intensity or asymmetric spectral shape in a variety of materials including charge transfer complex, graphene and transition metal oxides^29^,^30^,^31^. In the present system, the underlying electronic excitation around 20 meV is nothing but Drude response. Thus, the significant enhancement of spectral intensity for soft phonon can be attributed to the interaction with the Drude response, more specifically, the coupling between the polarization and conduction electrons. Incidentally, the spectral weight of soft phonon coupled to the Drude response may be related to that of the polaronic optical excitation appearing as the dopant induced in-gap state in the mid-infrared region (data not shown). The enhanced intensity of the former should be compensated by the reduction of the latter at low temperatures, while this sum rule cannot be clearly identified within our experimental accuracy. Although the direct interaction between conduction electrons and infrared active transverse phonon is usually negligible in centrosymmetric metals or semiconductors, it can be promoted by the broken inversion symmetry as in the present case. Indeed, within the scheme of quantum kinetic theory, Edelstein proposed that the transverse mode can mix with the longitudinal one in a medium with polar symmetry, that is, the fluctuation of the electric dipole of the transverse optical phonon can interact with the local fluctuation of conduction electron density via the electrostatic (Fröhlich) interaction^32^. Such an unusual coupling between the electric polarization and conduction electrons inherent to the polar crystallographic symmetry may be responsible for realizing the emergent ferroelectric structure compatible with metallic charge transport in this most canonical ferroelectrics.

To summarize, we demonstrated that an emergent polar metallic phase characterized by the nanometer-scale complex domain structure is invoked by doping the canonical ferroelectric BaTiO_3_ with conduction electrons. In this phase, the uniaxial anisotropy of polarization appears to be significantly reduced due to the competing ferroelectric phases with different polarization directions, reminiscent of the polar embryo structure in frustrated/relaxor ferroelectrics. Moreover, the spectral intensity of infrared soft phonon is remarkably enhanced in this metallic phase, suggesting the presence of unusual coupled dynamics between polarization and conduction electrons promoted by the broken inversion symmetry. The doped ferroelectrics may offer a fertile ground for research of the unique entanglement between the static/dynamic ferroelectric structure and charge transport phenomena via unusual electron-lattice coupling.

## Methods

### Crystal growth and transport measurements

The single crystalline samples of (Ba_0.97_Sr_0.03_)_0.98_La_0.02_TiO_3_ were grown by the floating zone method in Ar-atmosphere. Here, Sr is dilutely doped for a technical reason; a large-size single crystal can be acquired by avoiding the incorporation of hexagonal (non-perovskite) crystal structural phase during the crystal growth. The electrical or structural properties of the dilutely Sr-doped system is similar to those of Sr-free system. The quality of crystal was structurally characterized by the in-house x-ray diffraction. The resistivity and Hall resistivity was measured with use of the four-probe method using a commercial instrument (Physical Property Measuremet System, Quantum Design).

### X-ray scattering

The x-ray diffraction experiment was performed using imaging plate diffractometers and Si(111) monochromatized synchrotron radiation 35 keV and 24.7 keV at the beam lines 02B1 and 44B2 at SPring-8, respectively. The size of ferroelectric clusters is defined as ξ = FWHM^−1^, where FWHM (nm^−1^) denotes the full-width at half-maximum of the Lorentzian fit to the data. The spectra of inelastic x-ray scattering were acquired at beam line 43LXU at SPring-8. The data were collected using the Si (11 11 11) reflection at 21.748 keV. The typical momentum and energy resolution, ΔQ is (0.03 0.03 0.04) in reciprocal lattice unit and 1.8 meV, respectively.

### Transmission Electron Microscopy

The foil specimens for transmission electron microscopy (TEM) experiments were prepared by crushing the single crystal and mounting the fragments onto Cu grids. Convergent-beam electron diffraction patterns were obtained using an energy-filter TEM (JEM-2010FEF) at an accelerating voltage of 100 kV. Dark-feld TEM images were obtained using a conventional TEM (JEM-2100F) at an accelerating voltage of 200 kV. A liquid-nitrogen cooling holder was used to control temperature of specimens.

### Optical measurements

Temperature dependence of reflectivity spectra at nearly normal incidence was measured between room temperature and 10 K in the energy region of 5 meV–5 eV with polarized light. We used a Fourier transform spectrometer (grating-type monochromator equipped with a microscope) in the photon energy region of 0.008–0.7 eV (0.5–5 eV). In the region of 3–40 eV, we carried out the measurement at room temperature with use of synchrotron radiation at UV-SOR, Institute for Molecular Science, Japan. The optical conductivity spectra were derived on the basis of Kramers-Kronig analysis. The Hagen-Rubens extrapolation were assumed below 5 meV and *ω*^−4^-type extrapolation above 40 eV.

## Additional Information

**How to cite this article**: Fujioka, J. *et al.* Ferroelectric-like metallic state in electron doped BaTiO_3_. *Sci. Rep.* **5**, 13207; doi: 10.1038/srep13207 (2015).

## Supplementary Material

Supplementary Information

## Figures and Tables

**Figure 1:**
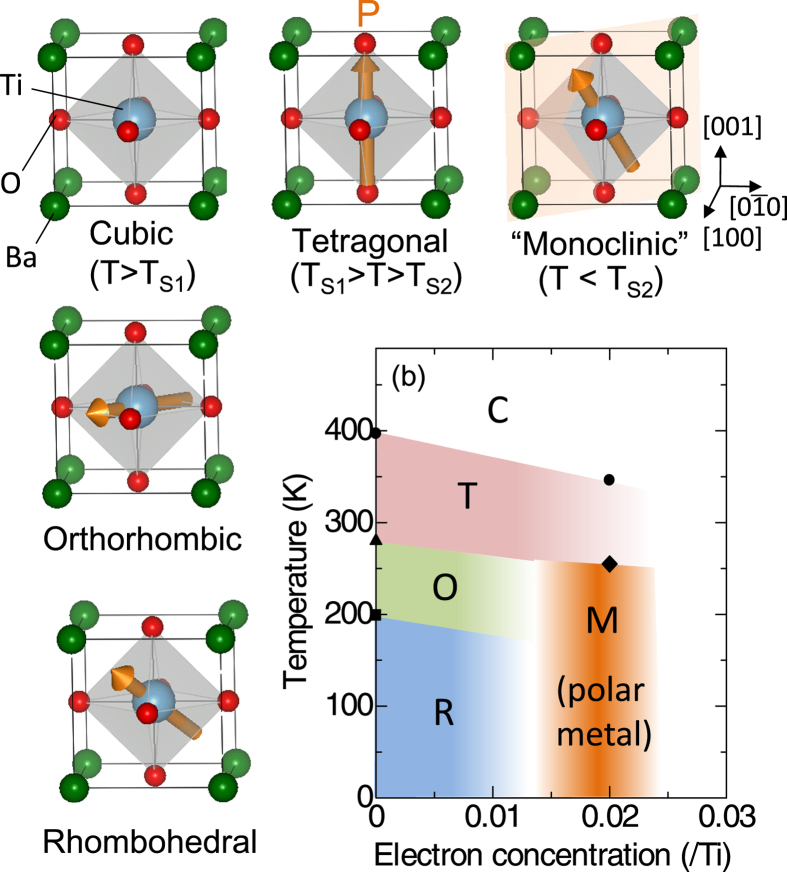
Structural phase diagram and schematic view of the macroscopic polarization in electron doped BaTiO_3_. C, T, O, R and M represent cubic, tetragonal, orthorhombic, rhombohedral and monoclinic, respectively. Arrows denote the direction of macroscopic electric polarization (*P*). *P* is pointing toward 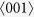, 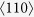 and 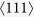 in tetragonal, orthorhombic and rhombohedral phase, respectively, while that in the ‘monoclinic’ phase does to a certain direction in-between 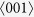 and 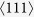 direction within 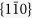 plane. The shaded plane indicates 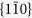 plane.

**Figure 2:**
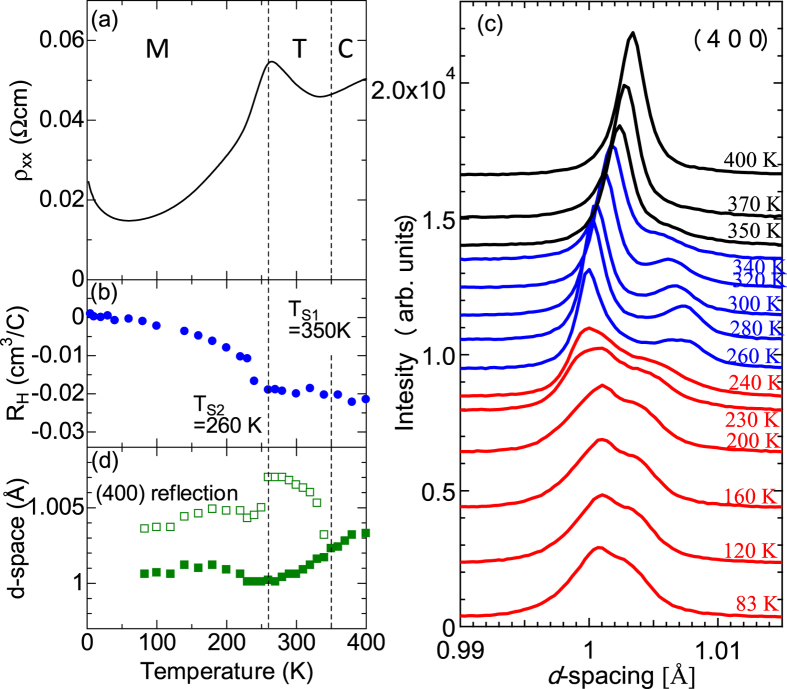
(**a**), (**b**) and (**d**) Temperature dependence of resistivity, Hall coefficient and *d*-spacing of (400) reflections, respectively. (**c**) The profile of fundamental Bragg reflection at (400) at various temperatures. Here the reflection index is denoted in cubic notation.

**Figure 3:**
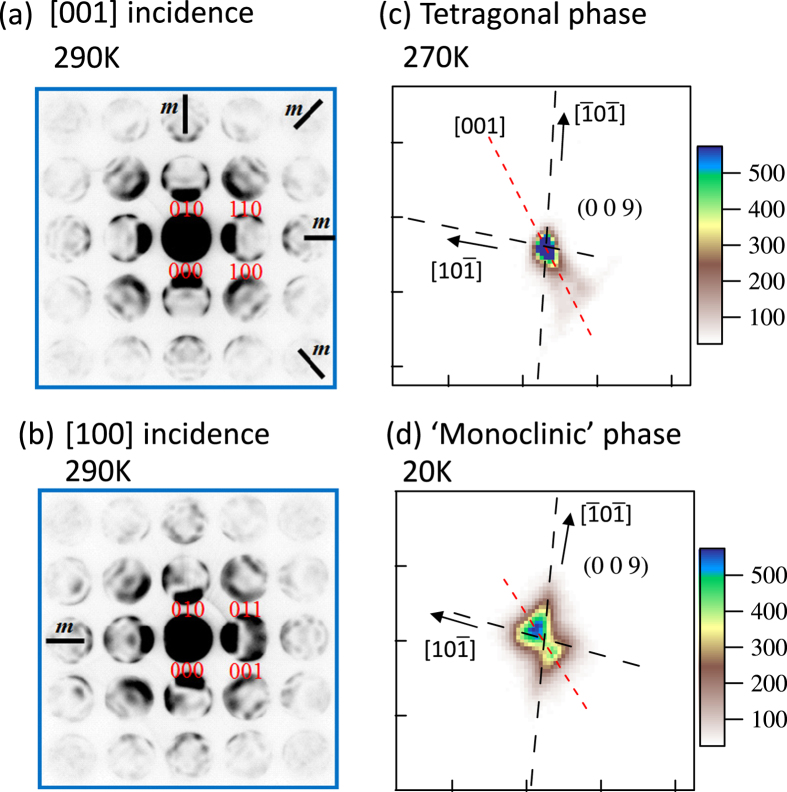
The patterns of convergent beam electron diffraction for the incident beams. (**a**) along [001] and (**b**) [100] axes at 290 K (tetragonal phase). The symmetries are 4*mm* and *m*, respectively. X-ray oscillation photographs around (009) reflection at (**c**) 270 K and (d) 20 K. The red dotted and black dashed lines denote the [001], 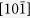 and 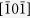 axis, respectively. The color-scale bar at right side of figure denotes the diffraction intensity in arbitrary units.

**Figure 4:**
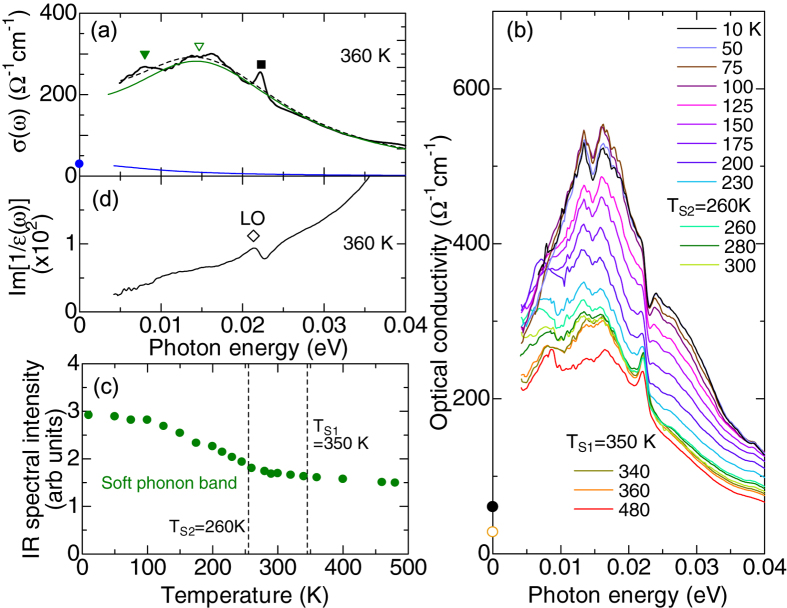
(**a**) Optical conductivity spectra at 360 K. The closed triangle, open triangle and closed square mark the polarization relaxation mode, Slater mode and Last mode, respectively. The thin blue, green and dashed lines denote the contribution from the Drude response, the damped harmonic oscillators coupled to Debye mode, and fitted spectra, respectively. (**b**) The optical conductivity spectra at various temperatures. The closed (open) circles mark the dc-conductivity at 10 K (360 K) deduced from the resistivity measurements. (**c**) Temperature evolution of the spectral intensity of soft phonon band. (**d**) The loss function Im[1/*ε*] spectra at 360 K. The diamond denotes the energy of longitudinal optical (LO) phonon.

**Figure 5:**
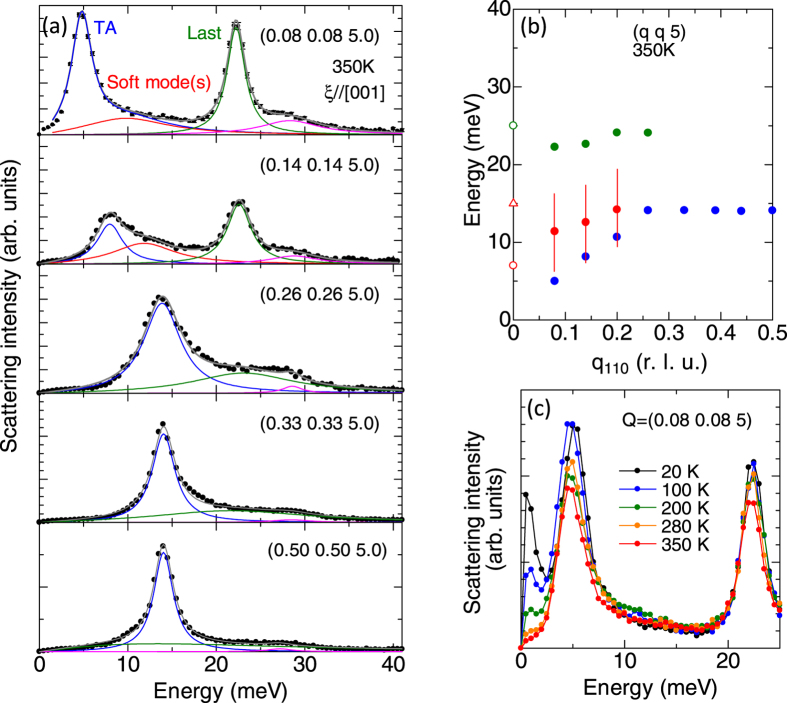
(**a**) Inelastic x-ray scattering spectra taken at 350 K. The blue, red and green lines denote the fitted results for transverse acoustic mode, soft mode and Last mode, respectively. The mode around 28 meV denoted by purple lines may be multi-phonon band or infrared inactive optical phonon. (**b**) Energy dispersion of phonons. The energies at *q* = 0 (open symbols) are determined by the optical conductivity spectra. (**c**) The inelastic scattering spectra at various temperatures at *Q* = (0.08 0.08 5). The peak-like structure below 3 meV is an artifact due to the Bose correction.

**Table 1:** The structural refinement results for tetragonal phase at 270 K, Space group P4mm (No. 99), lattice parameters *a* = *b* = 3.98350(10)Å, *c* = 4.00860(10)Å and *V* = 63.610Å^3^.

Atom	site	*x*	*y*	*z*	*U*_11_	*U*_22_	*U*_3_	*U*_12_	*U*_13_	*U*_2_
Ba, Sr, La	1a	0	0	0	0.0045	0.0045	0.00491(2)	0	0	0
Ti	1b	1/2	1/2	0.5145(2)	0.006250(10)	0.006250(10)	0.00330(9)	0	0	0
O (1)	2c	0		0.4933 1/2(4)	0.00557(5)	0.00675(6)	0.00780(10)	0	0	0
O (2)	1b	1/2	1/2	0.9863(4)	0.00667(6)	0.00667(6)	0.0081(2)	0	0	0

The reliability factors are *R* = 2.10%, *R*_w_ = 2.49%, GOF(Goodness of fit) = 1.038. Here the positions of Ba, La, and Sr ions are fixed. The atoms at site 1a is modeled by Ba. From this structure data, O-Ti-O angle is estimated as 175.13(12)°. In the tables, *x*, *y*, and *z* are the fractional coordinates. Anisotropic atomic displacement parameters are represented as *U*_11_, *U*_22_, *U*_33_, *U*_12_, *U*_13_, and *U*_23_ in units of (Å^2^).
