# Supplementary material for: Ferroelectric-like metallic state in electron doped BaTiO3
Source: Sci Rep. 2015 Aug 20;5:13207. doi: 10.1038/srep13207 (PMC4542543; doi:10.1038/srep13207)
Supplement: Supplementary Information [file srep13207-s1.pdf]

Supplementary Information for
Ferroelectric-like metallic state in electron doped $BaTiO_3$

J. Fujioka[1], A. Doi[1], D. Okuyama[2], D. Morikawa[2], T. Arima[2,3], K. N. Okada[1], Y Kaneko[2], T. Fukuda[4,5], H. Uchiyama[5,6], D. Ishikawa[5,6], A. Q. R. Baron[5,6], K. Kato[7], M. Takata[7] and Y. Tokura[1,2]

[1] *Department of Applied Physics and Quantum-Phase Electronics Center (QPEC), University of Tokyo, Hongo, Tokyo 113-8656, Japan*

[2] *RIKEN Center for Emergent Matter Science (CEMS), Wako 351-0198, Japan*

[3] *Department of Advanced Materials Science, University of Tokyo, Kashiwa 227-8561 Japan*

[4] *Syncrotron Radiation Research Unit, JAEA/SPring-8, Sayo, Hyogo 679-5148, Japan*

[5] *RIKEN SPring-8 Center, Sayo, Hyogo 679-5148, Japan*

[6] *Research and Utilization Division, JASRI/SPring-8, Sayo, Hyogo 679-5198, Japan*

[7] *Structural Materials Science Laboratory, RIKEN SPring-8 Center, Sayo, Hyogo 679-5148, Japan*

1. The crystal growth and analysis of crystal structure

The single crystalline samples of $(Ba_{0.97}Sr_{0.03})_{0.98}La_{0.02}TiO_3$ were grown by the floating zone method in Ar-atmosphere. Here, Sr is dilutely doped for a technical reason; a large-size single crystal can be acquired by avoiding the incorporation of hexagonal (non-perovskite) crystal structural phase during the crystal growth. The electrical or structural properties of the dilutely Sr-doped system is similar to those of Sr-free system. The quality of crystal was structurally characterized by the in-house x-ray diffraction.

Figure S1**a** shows the typical x-ray oscillation photograph nearby (009) reflection at 270 K. No superlattice reflections are discernible within the experimental accuracy. Given the crystallographic symmetry with P4mm space group, we refined the crystal structure on the basis of the collected data sets of synchrotron x-ray diffraction. The comparisons between observed and calculated structure factors are shown in Fig. S1**b**. 37081 reflections were observed, and 3426 of them were independent ($R_{\text{int}} = 0.0282$). 14 parameters were used for the refinement (Reflection/Parameter ratio is 245). The absolute structure was deduced based on Flack parameter, 0.77(4), refined using 115 Friedel pairs.

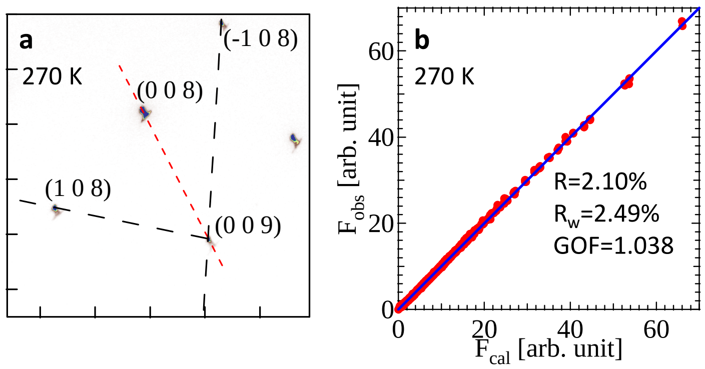

Figure S 1: **The single crystalline x-ray diffraction at 270 K**, The red dotted and black dashed lines denote the [001], [10$\bar{1}$] and [$\bar{1}$0$\bar{1}$] axis, respectively. Comparison of the calculated (F_{cal}) and the experimental (F_{obs}) structure factors at 270 K.

2. Transmission electron microscopy experiment in the "monoclinic" phase

Figure S2**a** shows the CBED pattern at 90 K with the [001] incidence. Here we denote both reflections and electron incidences in electron diffraction patterns in the cubic notation. In contrast with the CBED pattern for the tetragonal phase, the mirror and rotational symmetries are no longer discernible. More specifically, the CBED pattern is extremely sensitive to the beam position and we could not find the specimen area with clear mirror and rotational symmetries. Since the spot size of convergent beam is a few nanometer and typical specimen thickness is several tens to 150 nm, this conversely suggests that the crystal structure is heterogeneous in nanometer scale.

The nanometer scale domain structure is more clearly observed in the dark-field image. Figures S2**b-e** show the dark-field image of selected area at 90 K (monoclinic phase) as well as at 290 K (tetragonal phase), respectively. Images are taken using 011 reflection with the [001] incidence. In the raw images (Fig. S2**b** and **d**), the stripe patterns, which comes from the gradual change of specimen thickness, governs the contrast of image. For clarity, we have masked these long period structures with typical size larger than 25 nm in the Fourier transformed patterns (Fig. S2**c** and **e**). Spotty patterns with typical size of 10 nm corresponding to the nanometer scale polar domains are identified at 90 K, while one can identify nothing but a background noise at 290 K. This roughly agrees with the domain-size estimated from the x-ray diffuse scattering.

3. Modeling of low energy optical conductivity spectra

We have assumed the Drude response and damped harmonic oscillators coupled to the Debye mode to model the free carrier response and broad continuum band of soft mode, respectively. The optical conductivity spectra is modeled by following formula [1, 2],

$$\sigma(\omega) = \sigma_D(\omega) + \sigma_{\mathrm{ph}}(\omega) \tag{1}$$

$$\sigma_D(\omega) = \frac{\sigma_{dc}}{1+\omega^2\tau^2} \tag{2}$$

$$\sigma_{\mathrm{ph}}(\omega) = i\omega \mathrm{Im}[\epsilon_\infty + \Omega \cdot G(\omega) \cdot \Omega] \tag{3}$$

with the Green function $G(\omega)$ and array of mode plasma frequencies $\Omega = (0,\ \Omega_s)$. Specifically, $G(\omega)$ is defined by

$$G(\omega) = \begin{pmatrix} 1 - i\omega/\gamma_D & \delta_D \\ \delta_D & \omega_0^2 - \omega^2 - i\omega\Gamma_s \end{pmatrix}^{-1}, \tag{4}$$

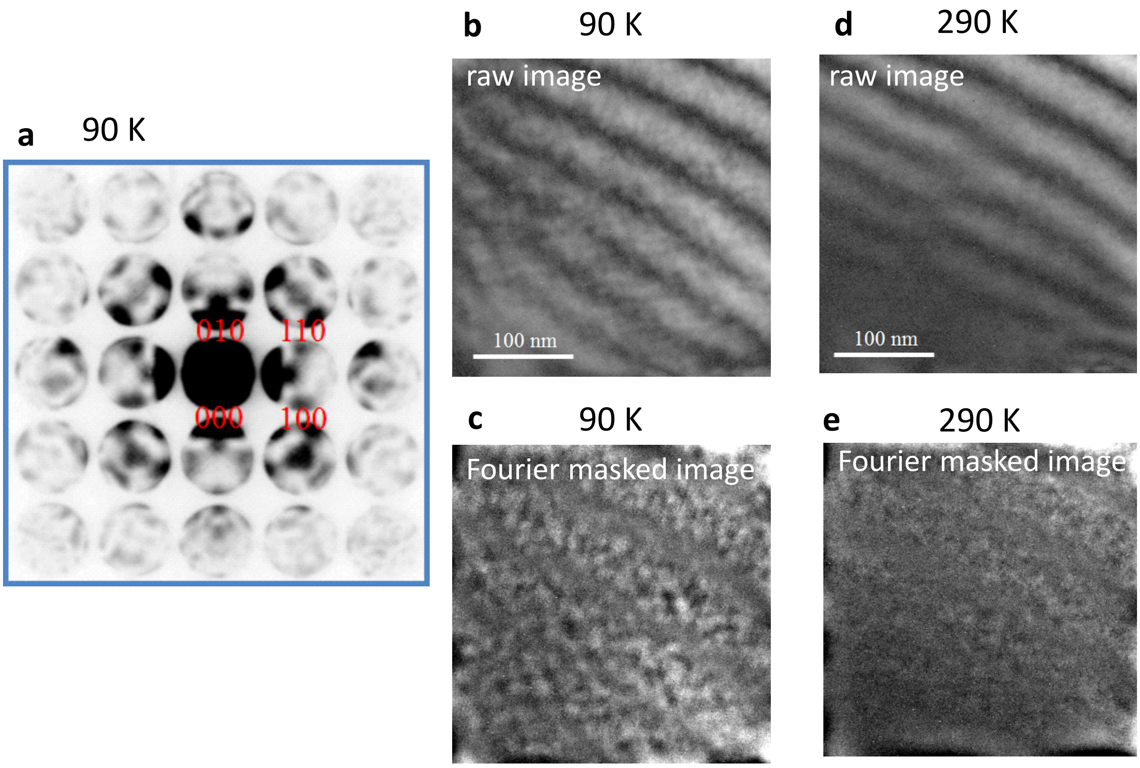

Figure S 2: **Transmission electron microscopy experiment** **a** The CBED pattern taken with [001] incidence at 90 K. **b-e** The dark-field images of selected area in the "monoclinic" phase at 90 K (**b,c**) and tetragonal phase at 290 K (**d,e**). The stripe patterns in **b, d** are interference fringe of electron beam, corresponding to the gradual change of specimen thickness. Here, **b,d** and **c,e** are raw images and inverse Fourier images after masking long period structure, respectively (see also text).

Here, ω_0, δ_D, and γ_D are eigen energy of Slater mode, magnitude of hybridization and damping rate of relaxation mode, respectively. We note that Ω_s represents the spectral intensity of the coupled mode. The integrated intensity of σ_{ph} is calculated as the spectral intensity for the soft phonon band.

---

[1] Shapiro, S. M., Axe. J. D., Shirane, G. and Riste, T. Critical Neutron Scattering in $SrTiO_3$ and $KMnF_3$ *Phys. Rev. B.* **6**, 4332 (1972).

[2] Hlinka, J. *et al.,* Coexistence of the Phonon and Relaxation Soft Modes in the Terahertz Dielectric Response of Tetragonal $BaTiO_3$ *Phys. Rev. Lett.* **101**, 167402 (2008).
